# Reevaluation of antibody-dependent enhancement of infection in anti-SARS-CoV-2 therapeutic antibodies and mRNA-vaccine antisera using FcR- and ACE2-positive cells

**DOI:** 10.1038/s41598-022-19993-w

**Published:** 2022-09-16

**Authors:** Jun Shimizu, Tadahiro Sasaki, Ritsuko Koketsu, Ryo Morita, Yuka Yoshimura, Ami Murakami, Yua Saito, Toshie Kusunoki, Yoshihiro Samune, Emi E. Nakayama, Kazuo Miyazaki, Tatsuo Shioda

**Affiliations:** 1 MiCAN Technologies Inc, KKVP 1-36, Goryo-ohara, Nishikyo-Ku, Kyoto, 615-8245 Japan; 2 Department of Viral Infections, Research Institute for Microbial Diseases, Osaka University, 3-1, Yamada-oka, Suita, Osaka 565-0871 Japan; 3 Osaka City Juso Hospital, 2-12-27, Nonakakita, Yodogawa-ku, Osaka, 532-0034 Japan

**Keywords:** SARS-CoV-2, RNA vaccines

## Abstract

Many therapeutic antibodies (Abs) and mRNA vaccines, both targeting SARS-CoV-2 spike protein (S-protein), have been developed and approved in order to combat the ongoing COVID-19 pandemic. In consideration of these developments, a common concern has been the potential for Ab-dependent enhancement (ADE) of infection caused by inoculated or induced Abs. Although the preventive and therapeutic effects of these Abs are obvious, little attention has been paid to the influence of the remaining and dwindling anti-S-protein Abs in vivo. Here, we demonstrate that certain monoclonal Abs (mAbs) approved as therapeutic neutralizing anti-S-protein mAbs for human usage have the potential to cause ADE in a narrow range of Ab concentrations. Although sera collected from mRNA-vaccinated individuals exhibited neutralizing activity, some sera gradually exhibited dominance of ADE activity in a time-dependent manner. None of the sera examined exhibited neutralizing activity against infection with the Omicron strain. Rather, some ADE of Omicron infection was observed in some sera. These results suggest the possible emergence of adverse effects caused by these Abs in addition to the therapeutic or preventive effect.

## Introduction

Therapeutic Ab drugs targeting SARS-CoV-2 S-protein have shown high preventive efficacy against disease development^1–3^. In addition, current SARS-CoV-2 vaccines for humans also target the S-protein on viruses as a critical antigen^4^. These vaccines generate robust neutralizing Abs^5–7^, but for both Ab drugs and vaccines targeting the S-protein, the possible induction of Ab-dependent enhancement (ADE) of infection is a concern^8–11^. Recent reports have demonstrated that neutralizing mAbs against S-protein can exhibit ADE activity in a limited window of Ab concentrations^12–14^. An important issue requiring reconsideration is that the cells used to evaluate ADE potential are different in each report. In many cases, Fc-receptor (FcR)-positive and angiotensin-converting enzyme 2 (ACE2, the major receptor for SARS-CoV-2^15–17^)-negative cells lines (Raji, THP-1, and K562) are used as host cells for infection of SARS-CoV-2 pseudo-viruses expressing S-protein or authentic SARS-CoV-2^12–14,18,19^. These reports have demonstrated that some anti-S protein mAbs have the potential to induce ADE of infection. The observed ADE can be blocked in the presence of FcR-blocker, demonstrating FcR dependence. Likewise, the Ab drugs casirivimab and imdevimab^1,20,21^, which target the SARS-CoV-2 S-protein, have also been evaluated by using FcR-positive and ACE2-negative cell lines (U937, THP-1, IM9, K562, and Raji)^22^. In this case, the report concluded that these mAbs have no ADE activity. In contrast, recent reports have demonstrated that some plasma samples from COVID-19 patients can enhance SARS-CoV-2 infection only in cells expressing both FcR and ACE2^23,24^. We also recently reported that ADE observed with sera from COVID-19 convalescents is FcR- and ACE2-dependent^25^. Therefore, current experimental conditions for evaluating ADE in vitro are inconsistent.

We have reported that human iPS cell-derived, immortalized, and ACE2- and TMPRSS2-expressing myeloid cell lines (Mylc cell lines) are highly susceptible to SARS-CoV-2 infection^25^. The infection of SARS-CoV-2 in Mylc cell lines was FcR- and ACE2-dependent. In the present study, we reevaluated whether the approved therapeutic Ab drugs (casirivimab, imdevimab, and sotrovimab^26,27^) have any potential to cause ADE even in FcR- and ACE2-positive cells. In addition, we investigated sera from mRNA (Moderna)-vaccinated individuals in terms of ADE-causing potential by using the same double-positive cells. Here, we show that the casirivimab and imdevimab mAbs have the ability to induce ADE, but sotrovimab does not. Furthermore, some sera from individuals vaccinated with the mRNA vaccine targeting the S-protein also exhibited ADE potential against infection with the original strain. All sera examined, including sera showing neutralizing activity against the original Wuhan strain of SARS-CoV-2, exhibited no neutralizing activity against Omicron. Rather, some ADE activity was observed in some sera.

## Results

**Figure 1:**
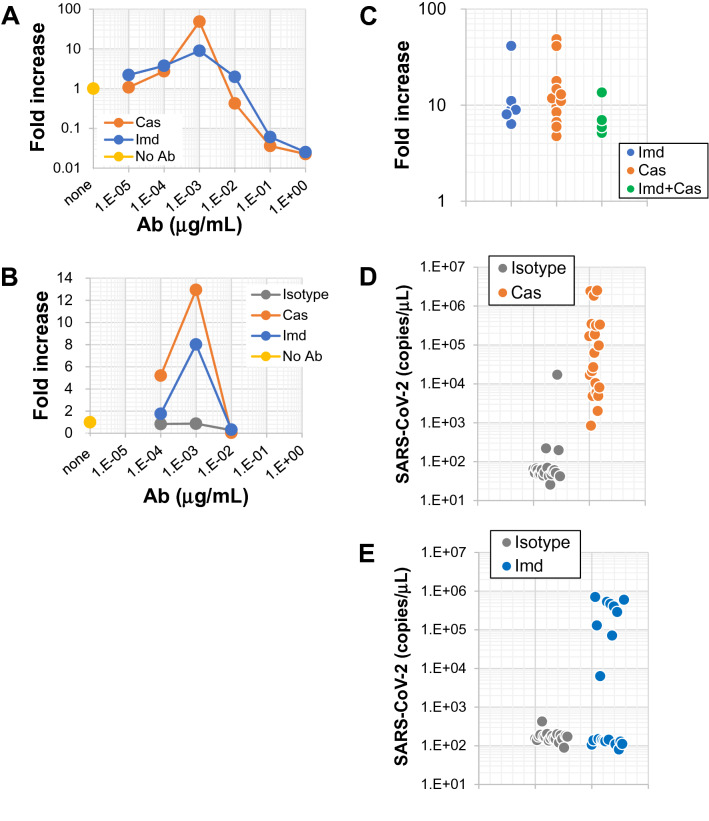
Anti-SARS-CoV-2 neutralizing mAbs, casirivimab and imdevimab, have the potential to cause ADE of infection. (**A**,**B**) Clone 35 cells (2 × 10^4^/well in a 96-well flat plate) were cultured with SARS-CoV-2 (original strain, 4 × 10^3^ copies/μL) in the presence (N = 3) or absence (N = 3–6, control culture) of serially-diluted Cas, Imd, or isotype control Abs as indicated. Three days later, the amount of virus in culture SNs was measured by qPCR. The increase of virus progeny in SNs is expressed as the fold increase compared with the amount of viruses cultured in the absence of Abs. (**C**) Clone 35 cells were cultured with SARS-CoV-2 in the presence or absence of a constant dose of the indicated Abs (final 1 ng/mL each) as in (**A**). Each symbol represents individual experiments. (**D**,**E**) Clone 35 cells were cultured as in (**A**), but with a low dose of SARS-CoV-2 (40 copies/μL). Each symbol represents individual wells (N = 20 per group).

Recent studies have shown in detail that monoclonal anti-S-protein Abs can function as ADE-causing Ab^12–14,18^. Some reports have demonstrated FcR-dependent ADE in the absence of ACE2, while others have found that both FcR and ACE2 are required for ADE^23–25^. The Ab drugs casirivimab and imdevimab (hereafter referred to as Cas and Imd) are anti-SARS-CoV-2 S-protein-neutralizing and therapeutic mAbs (human IgG_1_ isotype) that were approved after evaluation using many kinds of FcR-expressing cells (without ACE2 expression)^22^. First, we confirmed whether these mAbs have the potential to bind to FcR on cells. The cell line used in this assay is a Mylc line, K-ML2 (AT) clone 35 (hereafter, clone 35). Clone 35 cells are CD16 (FcγRIII) negative, and CD32 (FcγRII) and CD64 (FcγRI) positive (Supplemental Fig. 1). Clone 35 cells were stained with an irrelevant Ab, anti-human T-cell receptor (TCR) Ab (Supplemental Fig. 2). However, this staining was blocked in the presence of FcR-bindable Ab (4G2) as well as FcR-blocker, demonstrating that staining with the irrelevant anti-TCR Ab is FcR-dependent and nonspecific. In staining with the irrelevant anti-TCR Ab, we pre-added Cas or Imd Ab to the cells. This experiment revealed that Cas and Imd Abs can inhibit the staining of the irrelevant Ab as well as FcR-bindable Ab (4G2 mAb, Supplemental Fig. 2). These results demonstrate that Cas and Imd mAbs, as whole human IgG_1_ molecules, have the ability to bind to FcR, which prompted us to reevaluate if they have any ADE activity even on FcR- and ACE2-positive clone 35 cells. Clone 35 cells were cultured with a constant dose of authentic SARS-CoV-2 virus (original strain: 4,000 copies/μL) in the presence or absence of a titrated amount of Cas or Imd Abs (Fig. 1A). Three days later, the amounts of SARS-CoV-2 RNA in the culture supernatants (SNs) were examined by quantitative PCR (qPCR). At high doses of mAbs, significant neutralizing activities were observed (for example, more than 90% neutralization at 100 ng/mL, Fig. 1A). These neutralization efficiencies were consistent with those previously reported^22^, indicating that these mAbs in these experiments maintained their activities. However, at diluted concentrations (1 ng/mL final), both mAbs exhibited obvious ADE activity (Fig. 1A,B). The window of Ab concentration in which ADE was observed was narrow (Supplemental Fig. 3). Isotype-matched control Ab (human IgG_1_) had no ADE activity (Fig. 1B). This ADE was observed even with a mixture of these mAbs (Imd + Cas in Fig. 1C). In other sets of experiments, we cultured clone 35 cells with an extremely low dose of virus (40 copies/μL) in the presence of Cas, Imd mAb, or isotype control Ab at the same concentration (1 ng/mL) (Fig. 1D,E). At this low dose of virus, there was almost no significant viral expansion in the presence of isotype control Ab: no virus expansion in 19 wells (Fig. 1D) or 20 wells (Fig. 1E) out of 20 wells. However, the presence of a low amount of Cas or Imd mAb (1 ng/mL) augmented virus infection and expansion in all 20 individual wells (Fig. 1D, p = 0.026 between isotype and Cas cultures) or in 9 out of 20 wells (Fig. 1E, p = 0.005), respectively, suggesting that these mAbs have the potential to make cells more sensitive to infection. The ADE observed for clone 35 cells was not observed for the parental cells, K-ML2 cells lacking the expression of ACE2 and TMPRSS2 (Supplemental Fig. 4A), and was blocked in the presence of an FcR-bindable and competitive Ab (4G2 mAb) (Supplemental Fig. 4B,C). Moreover, ADE of infection was also observed with the SARS-CoV-2 Delta strain (Supplemental Fig. 4D) and for other Mylc cell lines (D05 and PhF cell lines) (Supplemental Fig. 4E). Taken together, these results demonstrate that Cas and Imd mAbs have the potential to cause ADE of infection at a particular Ab concentration, and that the ADE observed is dependent on ACE2 and FcR.

**Figure 2:**
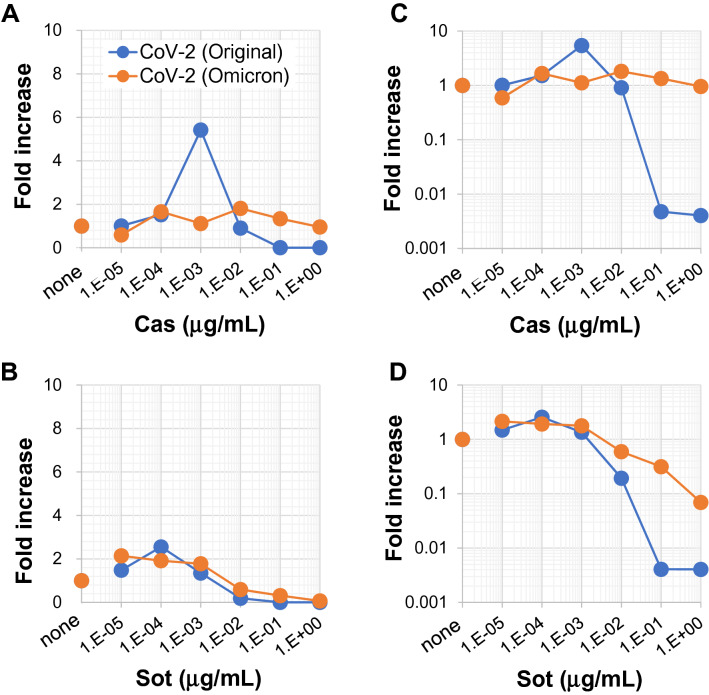
Sotrovimab mAb has no potential to cause ADE of infection. Clone 35 cells (2 × 10^4^/well in a 96-well flat plate) were cultured with SARS-CoV-2 (original or Omicron strain) in the presence (N = 3) or absence (N = 3–6, control culture) of serially-diluted Cas (**A**,**C**) or Sot (**B**,**D**) mAb. Three days later, the amount of virus in culture SNs was measured by qPCR. The increase of virus progeny in SNs is expressed as the fold increase compared with the amount of viruses cultured in the absence of mAb on a linear scale (**A**,**B**) or log scale (**C**,**D**).

Since the emergence and identification of the new Omicron variant of SARS-CoV-2^28^, the reactivity of the approved and neutralizing mAbs against SARS-CoV-2 Omicron has been investigated^29^. The Omicron strain is recognized by Cas mAb with extremely poor efficiency and not at all by Imd mAb. In contrast, another mAb, sotrovimab (Sot)^26,27^, has retained neutralizing activity against Omicron^29^. Sot mAb is also an anti-S-protein mAb with a human IgG_1_ backbone, the same as Cas and Imd mAbs, and contains a two amino acid modification in the Fc region^26,27^. We then evaluated whether Sot mAb has the potential to cause ADE in our experimental system. Sot mAb was able to bind to FcR as well as Cas mAb (Supplemental Fig. 5). However, in contrast to Cas mAb (Fig. 2A,C), Sot mAb exhibited no ADE activity at any Ab concentration examined and had neutralizing activity at a higher Ab concentration against both the original and Omicron strains (Fig. 2B,D). Cas mAb had no neutralizing activity against Omicron even at higher Ab concentrations (Fig. 2C). These results are consistent with previous reports^26,29^, demonstrating that our experimental system using clone 35 cells is capable of evaluating both neutralizing and ADE activity, and supports the hypothesis that Sot mAb is a neutralizing Ab with no ADE potential.

**Figure 3:**
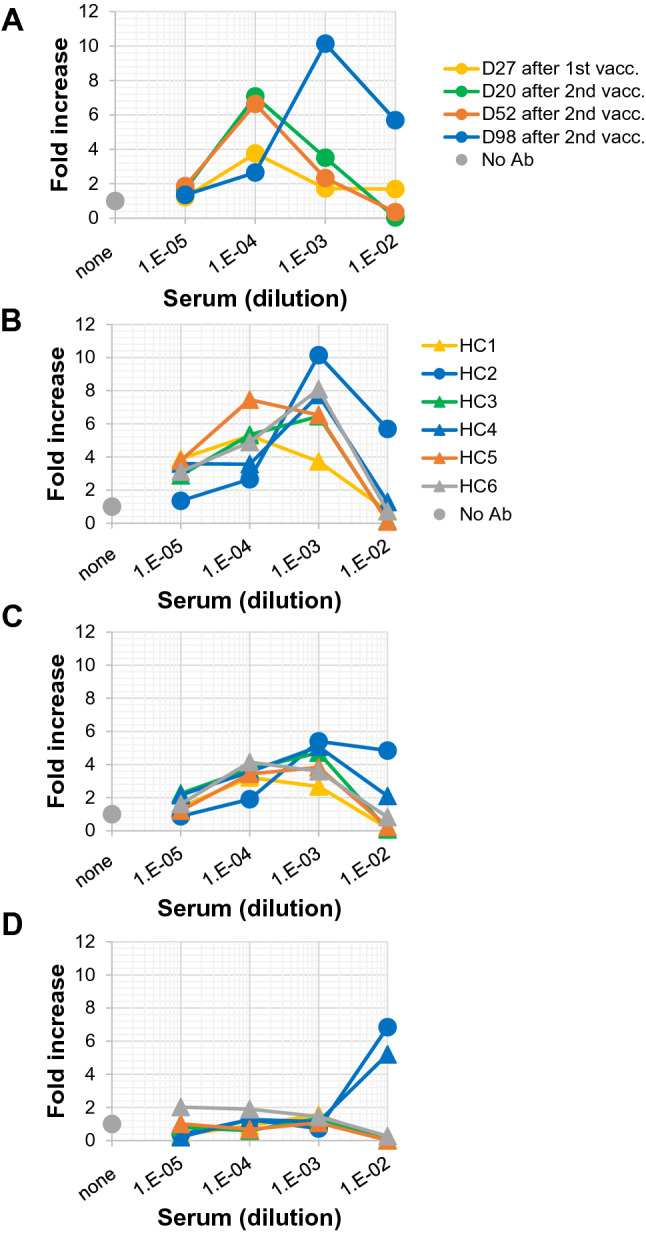
ADE potential in sera collected from mRNA-vaccinated volunteers. (**A**) Clone 35 cells (2 × 10^4^/well in a 96-well flat plate) were cultured with SARS-CoV-2 (original strain, 4 × 10^3^ copies/μL) in the presence (N = 3) or absence (N = 3–6, control culture) of serially-diluted sera from the same individual (HC2). Sera were collected at different time points after the first vaccination (on day 27) and second vaccination (on days 20, 52, and 98) as indicated in the insert. (**B**–**D**) Sera were collected on day 98 (**B**), day 133 (**C**), and day 175 (**D**) after the second vaccination from six healthy control volunteers (HC1-6, see insert in (**B**)) and added to the cultures as in (**A**). Three days later, the amount of virus in culture SNs was measured by qPCR. The increase of virus progeny in SNs is expressed as the fold increase compared with the amount of viruses cultured in the absence of serum on a linear scale (this figure) or log scale (Supplemental Fig. 6).

We have shown that anti-SARS-CoV-2 S-protein neutralizing mAbs as whole molecules (human IgG_1_) can function as ADE-causing Ab (Fig. 1). These results raise the possibility that SARS-CoV-2 mRNA vaccines targeting the S-protein also induce ADE-causing Abs as well as neutralizing Abs. Next, we examined whether sera from mRNA (Moderna)-vaccinated volunteers have neutralizing or ADE activities, how long these activities last, and how they change in a time-dependent manner. Clone 35 cells were cultured with authentic SARS-CoV-2 virus (original strain) along with or without titrated sera from the same volunteer (HC2, Fig. 3A). Neutralizing activity was not detected in serum collected on day 27 after the first vaccination, but was detected at the highest concentration (1/100 dilution) of serum collected on days 20 and 52 after the second vaccination (Fig. 3A and Supplemental Fig. 6A). Simultaneously, obvious ADE activity was also detected at a diluted concentration (1/10,000 dilution) of serum. Importantly, serum collected on day 98 after the second vaccination exhibited no neutralizing activity at all under the serum dilutions examined, but maintained clear ADE activity (Fig. 3A). Sera from six individuals (including the same individual, HC2, shown in Fig. 3A) on day 98 after the second vaccination exhibited either neutralizing activity (HC3 and 5, Fig. 3B and Supplemental Fig. 6B) or no neutralizing activity (HC1, 2, 4, and 6, Fig. 3B and Supplemental Fig. 6B). However, in all these sera examined, ADE activity was detected to a greater or lesser degree (Fig. 3B). Sera collected on day 133 (Fig. 3C) after the second vaccination maintained almost the same pattern with the results on day 98 (Fig. 3B). On day 175 (Fig. 3D), ADE activity was observed only at the highest concentration in some sera, but with a relatively low magnitude. Some sera still maintained neutralizing activity at the highest concentration of serum (1/100 dilution, Supplemental Fig. 6D). Taken together, these results demonstrate that after vaccinations, neutralizing Abs are induced and persist for a long time in some individuals, but ADE-causing Abs also exist from the early stage and persist for a longer period than do neutralizing Abs in some individuals (HC2 and HC4 in Fig. 3B–D). It is noteworthy that ADE observed at a higher concentration of serum, that is at low dilution (1/100), might mean a more vulnerable stage in terms of susceptibility to infection, because no neutralizing activity was detected.

Finally, we examined the effect of sera after vaccination against infection with the SARS-CoV-2 Omicron strain. Clone 35 cells were cultured in the presence of sera collected before vaccination (Supplemental Fig. 7A) or on day 175 after the second vaccination (Supplemental Fig. 7B) along with authentic SARS-CoV-2 Omicron virus. Although some sera maintained neutralizing activity against the original strain (Supplemental Fig. 6D) even on day 175 after the second vaccination, none of these sera had neutralizing activity against Omicron (Supplemental Fig. 7B). Rather, one serum (HC6) exhibited some ADE activity (Supplemental Fig. 7B). The observed augmentation in infection with Omicron was serum dose-dependent and required ACE2, because the parental cell line (K-ML2, lacking ACE2 and TMPRSS2) of clone 35 exhibited no infection with Omicron even in the presence of serum (Supplemental Fig. 8). These results suggest that the rapid spread of Omicron around the world may in part result from the lack of cross-neutralization against Omicron and some ADE activity of sera after vaccination.

## Discussion

Recent studies have demonstrated several mechanisms leading to ADE, which can be FcR-dependent but ACE2-independent, FcR-independent but ACE2-dependent, S-protein conformational change-dependent, or both FcR- and ACE2-dependent ADE^12,23–25,30^. In this study, we demonstrated that an FcR- and ACE2-positive Mylc cell line can be utilized for identifying and studying ADE mediated by different mechanisms.

Sera collected after mRNA vaccination targeting the S-protein in SARS-CoV-2 had the potential to cause ADE (Fig. 3). Even from an early stage after vaccination, ADE potential was detected as well as neutralizing activity, showing neutralization at a high concentration of serum and ADE at a low concentration (Fig. 3A). Our experiments using anti-SARS-CoV-2 neutralizing mAbs (Cas and Imd) and other reports^12–14^ have demonstrated that these opposing activities (neutralization and ADE) are exhibited by the same Ab (Fig. 1A). Therefore, although serum after vaccination contains various anti-S-protein Abs, the neutralization and ADE observed in sera after vaccination might result, in part, from the same Abs. Here, it is noteworthy that ADE was observed only within a relatively narrow window of Ab and serum concentrations (Fig. 3 and Supplemental Fig. 3). Under these limited Ab concentration conditions, the amount of virus added to the culture seems to be unrelated to the development of ADE. The presence of mAbs with ADE potential at 1 ng/mL indeed enhanced the efficiency of infection even with an extremely low dose of virus (40 copies/μL, Fig. 1D,E) as well as at 4,000 copies/μL (Fig. 1A–C). Taken together, these results suggest that the concentration of Ab is more critical than the amount of virus in inducing either neutralization or ADE as a whole. In addition, we need to realize that the efficiency in enhancement of infection ranged from a more than 10^4^-fold to a 10^1^-fold increase compared with that by an isotype-matched control Ab (Fig. 1D), and that the enhancement was observed in approximately half of the wells examined (9 out of 20 wells, Fig. 1E). Thus, these results also suggest that the concentration of ADE-causable Ab is not the only critical factor that results in the development of ADE. Furthermore, it should be re-emphasized that in our previous study, we observed that more than half of serum samples (51 out of 100 serum samples) from severe COVID-19 patients had no ADE-causable activity under the titrated serum concentration (10^–2^ to 10^–8^ final dilution), although all samples contained anti-S-protein Abs^25^. It is thus important to identify factor(s) other than Ab concentration involved in the occurrence of ADE.

It has been reported that Cas and Imd mAb reach a peak concentration (192 μg/mL and 198 μg/mL, respectively) immediately following intravenous administration of a single dose (600 mg each), and 46.2 μg/mL and 38.5 μg/mL on day 28 after dosing, respectively^22^, demonstrating a time-dependent decrease after intravenous administration. The results shown in Fig. 1 and Supplemental Fig. 3 indicate that ADE caused by these mAbs is observed at a narrow window of Ab concentrations (ADE peak at approximately 1 ng/mL of mAbs). Therefore, it is plausible that it takes a long time to reach the ADE-causable Ab concentration range, and in many cases, almost all viruses will be gone before reaching the unfavorable Ab concentration. Currently, it is important to pay attention to the possible adverse effects by the remaining or diminishing anti-SARS-CoV-2 Abs.

It has also been reported that neutralizing Abs induced after mRNA vaccinations decrease in a time-dependent manner^31^. Accordingly, we observed diminishing neutralizing activity in some vaccinated individuals in a time-dependent manner (Fig. 3A and Supplemental Fig. 6A). Simultaneously, the dominant ADE activity was observed at high concentrations of serum. This situation, i.e., observed ADE without neutralization at a low dilution of serum, is unfavorable for protection against infection. However, it must be remembered that, in contrast to the above-mentioned Ab-involved adverse events, protective cellular immunity is also induced and is involved in anti-SARS-CoV-2 responses in vivo^32^. Therefore, as a whole, this Ab-mediated adverse potential during virus expansion and the opposing protective effects by T-cell immunity might make it more difficult to recognize the involvement of ADE in SARS-CoV-2 reinfection or resurrection in vivo^12^. Six volunteers treated with the mRNA vaccine in this study maintained an uninfected state for approximately 8 months after the second vaccination, and also had a third vaccination. Therefore, there is no suggestive information between the data based on in vitro analyses (Fig. 3, Supplemental Figs. 6 and 7) and the clinical observations. To make clear the relationship between clinical variations and ADE observed in in vitro analyses, further investigations using a larger number of samples will be required.

The Omicron strain has been found to have many mutations in the S-protein^33,34^. These mutations result in reduced or zero effectiveness of Cas and Imd mAbs against the Omicron strain^29,35^. We also confirmed this in our experimental setting (Fig. 2). In addition to changes in the reactivity of mAbs to Omicron, we observed different behavior of Omicron in the ADE assay compared with the SARS-CoV-2 original strain (Supplemental Figs. 6D and 7B). Some sera from mRNA-vaccinated volunteers (collected on day 175 after the second vaccination) maintained neutralizing activity against the SARS-CoV-2 original strain (Supplemental Fig. 6D). In contrast, none of these sera exhibited neutralization, and some of them caused enhancement of infection with Omicron (Supplemental Fig. 7B). These results demonstrate that Abs raised by double vaccination (at least on day 175 after the second vaccination, Supplemental Fig. 7B) are less effective against Omicron as reported^36^, and suggest that the Omicron strain has acquired the ability to escape attack by pre-existing anti-SARS-CoV-2 Abs and in part can utilize infection-enhancing mechanisms, possibly including ADE, as a means of survival. At this time, we need to further investigate what the critical factors are that are included in sera and lead to the enhancement of Omicron infection. Although Omicron has many amino acid substitutions in the receptor-binding domain of the S-protein^33,34^, the receptors (ACE2) on target cells seem to still be required for infection (Supplemental Fig. 8). In fact, it has been reported that Omicron S-protein has high affinity for ACE2^35^. The unique mechanisms of Omicron infection, such as altered TMPRSS2 usage^35^, still need to be elucidated, and these unique features can be utilized in the development of new drugs.

Regarding neutralizing Ab drugs for SARS-CoV-2, it is important to avoid ADE potential. To this end, one promising approach is to modify the Fc-region in Abs to abrogate their binding to FcR^8,13,14,37^. The three therapeutic mAbs used in this study (Cas, Imd, and Sot) have human IgG_1_ backbones, Cas and Imd with no modification in the Fc region, and Sot with a two amino acid modification in the Fc region that confers extended half-life. All three of these mAbs bound to FcR irrespective of modification in the Fc region (Supplemental Figs. 2 and 5). Cas and Imd had ADE-causing potential (Fig. 1), while Sot had no ability to induce ADE (Fig. 2), demonstrating that an FcR-bindable Ab is not always an ADE-causing Ab. In addition, regarding the target epitope, Cas and Imd mAbs bind to distinct and non-overlapping regions of the receptor-binding domain (RBD) of the S-protein of SARS-CoV-2^21^. In contrast, Sot mAb targets an epitope on the S-protein that does not compete with ACE2 binding, which is a non-RBD region^26^. Therefore, taken together, it is not currently clear what critical factors are responsible for the lack of ADE in the case of Sot mAb, that is, the target epitope, modification of Fc, or both. These results suggest complex and multiple mechanisms for ADE. In any case, both FcR- and ACE2-positive Mylc cells will be useful for identifying ADE potential by Abs and antisera.

## Methods

### Mylc cell lines for SARS-CoV-2 infection

The procedure used to generate Mylc lines was previously reported^38,39^. Briefly, immortalized myeloid cell lines were established by the lentivirus-mediated transduction of cMYC, BMI-1, GM-CSF, and M-CSF into human iPS cell-derived myeloid cells. These cell lines were further induced to express ACE2 and TMPRSS2 using lentiviral vectors. The established cell lines (K-ML2(AT), D05, and PhF lines) were derived from different human iPS cells and maintained as bulk cell lines. In some experiments, cloned cells (clone 35) were established from bulk lines by limiting dilution. Each cell line was cultured in MEM alpha (Gibco) supplemented with 10% (vol/vol) fetal bovine serum under 37 °C, 5% CO_2_, and water-saturated humidity conditions.

### Viruses

SARS-CoV-2/Hu/DP/Kng/19-020 (original strain, GenBank accession number: LC528232.1) was provided by the Kanagawa Prefectural Institute of Public Health, Kanagawa, Japan, and the Omicron strain (hCoV-19/Japan/TY38-873/2021) by the National Institute of Infectious Diseases, Japan. SARS-CoV-2 Delta strain (hCoV-19/USA/PHC658/2021, NR-55611) was obtained from BEI Resources. SARS-CoV-2 viruses were passaged in VeroE6-TMPRSS2 cells (obtained from the National Institutes of Biomedical Innovation, Health, and Nutrition, JCRB cell bank, Japan) two times. Viral stocks were aliquoted, examined for the presence of viral RNA by quantitative RT-PCR, and tested for mycoplasma (they were mycoplasma-free) before being used for experiments. All experiments using live SARS-CoV-2 followed the approved operating procedures of the biosafety level 3 facility at the Research Institute for Microbial Diseases of Osaka University.

### Immunostaining

The following Abs were used: PE-conjugated anti-human CD16, CD32, CD64, and TCR Vβ8 Ab (mouse IgG_2a_) (all purchased from BioLegend); and PE-conjugated mouse IgG_1_ and mouse IgG_2b_ as isotype control Abs (eBioscience). For flow cytometry, cells were resuspended in Fc blocking buffer (1:500 dilution, eBioscience) and incubated on ice. Twenty minutes later, without washing, cells were stained with each Ab. The stained cells were analyzed by flow cytometry.

### In vitro infection of cells

Mylc cell lines (2 × 10^4^/well) were cultured with SARS-CoV-2 virus in 96-well flat-bottomed plates. Viral concentrations in culture SNs after three days of culture were determined by qRT-PCR. Total RNA was extracted from culture SNs using the QIAamp viral RNA mini kit (QIAGEN) following the manufacturer’s instructions. The primers used for qRT-PCR were as follows: SARS-CoV-2 forward, AGCCTCTTCTCGTTCCTCATCAC; reverse, CCGCCATTGCCAGCCATTC. The fold increases in viral quantity were calculated as follows: the fold increase = (virus concentration (copies/μL) of the experimental group with Ab)/(virus concentration (copies/μL) of the background control culture without Ab).

### ADE assay

Serum samples were obtained from SARS-CoV-2 mRNA vaccine (Moderna)-inoculated volunteers. Informed consent was obtained from all subjects involved in this study. The Institutional Review Board of the Research Institute for Microbial Diseases, Osaka University, approved the collection and use of sera from uninfected volunteers in this study (approval number: RIMD-2021-9). All experiments using materials from humans followed the approved operating procedures and guidelines at the Research Institute for Microbial Diseases of Osaka University. These sera were used after heat inactivation at 56 °C for 30 min. In ADE assays, viruses were first mixed with serially-diluted serum for 10 min at 37 °C, then cells were added to the mixtures. Cells cultured along with viruses in the absence of Abs were used as a background control. In some experiments, 4G2 Ab (mouse IgG_2a_, flavivirus group cross-reactive, purchased from American Type Culture Collection, Manassas, VA), human IgG_1_ isotype control Ab (BioLegend), and casirivimab, imdevimab, and sotrovimab (vials containing the leftover mAbs after clinical use were kept under 4 °C and kindly provided by Osaka City Juso Hospital under hospital permission) were used.

### Statistical analysis

The statistical significance of differences was evaluated with the Mann–Whitney test. Probability (p) less than 0.05 was considered significant.

## Supplementary Information

Supplementary Figures.

## Data Availability

All data generated or analyzed during this study are included in this article and its supplementary information files.
