# Author Correction: Reevaluation of antibody-dependent enhancement of infection in anti-SARS-CoV-2 therapeutic antibodies and mRNA-vaccine antisera using FcR- and ACE2-positive cells

**DOI:** 10.1038/s41598-023-28371-z

**Published:** 2023-01-20

**Authors:** Jun Shimizu, Tadahiro Sasaki, Ritsuko Koketsu, Ryo Morita, Yuka Yoshimura, Ami Murakami, Yua Saito, Toshie Kusunoki, Yoshihiro Samune, Emi E. Nakayama, Kazuo Miyazaki, Tatsuo Shioda

**Affiliations:** 1 MiCAN Technologies Inc, KKVP 1-36, Goryo-ohara, Nishikyo-Ku, Kyoto, 615-8245 Japan; 2 Department of Viral Infections, Research Institute for Microbial Diseases, Osaka University, 3-1, Yamada-oka, Suita, Osaka 565-0871 Japan; 3 Osaka City Juso Hospital, 2-12-27, Nonakakita, Yodogawa-ku, Osaka, 532-0034 Japan

Correction to: *Scientific Reports* 10.1038/s41598-022-19993-w, published online 16 September 2022

The original version of this Article contained an error, where the mention of “mRNA” was inappropriate.

As a result, in the Introduction,

“Therapeutic Ab drugs targeting SARS-CoV-2 S-protein have shown high preventive efficacy against disease development^1,2,3^. In addition, current SARS-CoV-2 mRNA vaccines for humans also target the S-protein on viruses as a critical antigen^4^. These mRNA vaccines generate robust neutralizing Abs^5,6,7^, but for both Ab drugs and vaccines targeting the S-protein, the possible induction of Ab-dependent enhancement (ADE) of infection is a concern^8,9,10,11^.”

now reads:

“Therapeutic Ab drugs targeting SARS-CoV-2 S-protein have shown high preventive efficacy against disease development^1,2,3^. In addition, current SARS-CoV-2 vaccines for humans also target the S-protein on viruses as a critical antigen^4^. These vaccines generate robust neutralizing Abs^5,6,7^, but for both Ab drugs and vaccines targeting the S-protein, the possible induction of Ab-dependent enhancement (ADE) of infection is a concern^8,9,10,11^.”

The original Article has been corrected.

