# Supplementary material for: Reevaluation of antibody-dependent enhancement of infection in anti-SARS-CoV-2 therapeutic antibodies and mRNA-vaccine antisera using FcR- and ACE2-positive cells
Source: Sci Rep. 2022 Sep 16;12:15612. doi: 10.1038/s41598-022-19993-w (PMC9481526; doi:10.1038/s41598-022-19993-w)
Supplement: Supplementary file 1 — Supplementary Figures. [file 41598_2022_19993_MOESM1_ESM.pdf]

Supplemental information

Reevaluation of antibody-dependent enhancement of infection in anti-SARS-CoV-2 therapeutic antibodies and mRNA-vaccine antisera using FcR- and ACE2-positive cells

Jun Shimizu[1], Tadahiro Sasaki[2], Ritsuko Koketsu[2], Ryo Morita[3], Yuka Yoshimura[1], Ami Murakami[1], Yua Saito[1], Toshie Kusunoki[1], Yoshihiro Samune[2], Emi E. Nakayama[2], Kazuo Miyazaki[1,*], Tatsuo Shioda[2,*]

[1] MiCAN Technologies Inc., KKVP 1-36, Goryo-ohara, Nishikyo-Ku, Kyoto 615-8245, Japan

[2] Department of Viral Infections, Research Institute for Microbial Diseases, Osaka University, 3-1, Yamada-oka, Suita, Osaka 565-0871, Japan

[3] Osaka City Juso Hospital, 2-12-27, Nonakakita, Yodogawa-ku, Osaka 532-0034, Japan

[*]Correspondence should be addressed to K.M. (kmiyazaki@micantechnologies.com) or T.S. (shioda@biken.osaka-u.ac.jp)

Supplemental Figure 1

Expression of FcR on clone 35 cells.

Clone 35 cells were stained with PE-conjugated isotype control Abs (red) or anti-CD16, CD32, or CD64 Abs (blue) in the presence of Fc-blocker. Data analysis was performed with FlowJo software.

Supplemental Figure 2

Casirivimab and imdevimab mAbs can bind to FcR.

Clone 35 cells were stained with PE-conjugated anti-human TCR Vβ8 Ab (mouse IgG_{2a}, BioLegend) in the presence or absence of the indicated Abs or reagent (blue). As a negative control, unstained cells were used (red).

Supplemental Figure 3

ADE induced by anti-SARS-CoV-2 neutralizing mAb is observed in a narrow window of Ab concentrations.

Clone 35 cells (2×10^4/well in a 96-well flat plate) were cultured with SARS-CoV-2 (original strain, 4.0×10^3 copies/μL) in the presence (N=3) or absence (N=3–6, control culture) of serially-diluted Cas mAb. Three days later, the amount of virus in culture SNs was measured by qPCR.

The increase of virus progeny in SNs is expressed as the fold increase compared with the amount of viruses cultured in the absence of mAb.

Supplemental Figure 4

ADE observed with anti-SARS-CoV-2 neutralizing mAbs is dependent on ACE2 and FcR.

(A) K-ML2 cells (ACE2- and TMPRSS2-negative cell lines) were cultured with SARS-CoV-2 in the presence or absence of serially-diluted Abs as in Fig. 1. (B and C) Clone 35 cells were cultured with SARS-CoV-2 (original strain) in the presence or absence of serially-diluted 4G2 Ab as an FcR-bindable competitive Ab and a constant dose (1 ng/mL) of Cas (B) or Imd (C) Abs. (D) Clone 35 cells were cultured with SARS-CoV-2 (original or Delta strains) in the presence or absence of serially-diluted Abs (one-to-one mixture of Imd and Cas Abs). The increase of virus progeny in SNs after three days of culture is expressed as the fold increase compared with the amount of viruses cultured in the absence of Abs. (E) Clone 35, D05, or PhF cell lines, all expressing ACE2 and TMPRSS2, were cultured with SARS-CoV-2 (original strain) in the presence or absence of Cas mAb (1 ng/mL). Three days later, the amount of virus in culture SNs was measured by qPCR. Error bars indicate SD; N=3.

Supplemental Figure 5

Sotrovimab mAb can bind to FcR.

Clone 35 cells were stained with PE-conjugated anti-human TCR Vβ8 Ab (mouse IgG_{2a}, BioLegend) in the presence or absence of the indicated mAbs (blue). As a negative control, unstained cells were used (red).

Supplemental Figure 6

ADE potential in sera collected from mRNA-vaccinated volunteers.

The result in Fig. 3 is shown on a log scale in the y-axis.

Supplemental Figure 7

Sera from mRNA-vaccinated volunteers have no neutralizing effect against Omicron infection.

Clone 35 cells (2×10^4/well in a 96-well flat plate) were cultured with SARS-CoV-2 (Omicron strain) in the presence (N=3) or absence (N=3–6, control culture) of serially-diluted sera from pre-vaccination (A) or post-vaccination day 175 (B) subjects. Three days later, the amount of virus in culture SNs was measured by qPCR. The increase of virus progeny in SNs is expressed as the fold increase compared with the amount of viruses cultured in the absence of serum.

Supplemental Figure 8

ACE2-dependent infection with the Omicron strain.

Clone 35 cells or parental K-ML2 cells (2×10^4/well in a 96-well flat plate) were cultured with SARS-CoV-2 (Omicron strain) in the presence (N=3) or absence (N=3–6, control culture) of serially-diluted serum from a vaccinated volunteer (HC1 in Fig. 3C). Three days later, the amount of virus in culture SNs was measured by qPCR and expressed on a linear scale (A) or log scale (B).

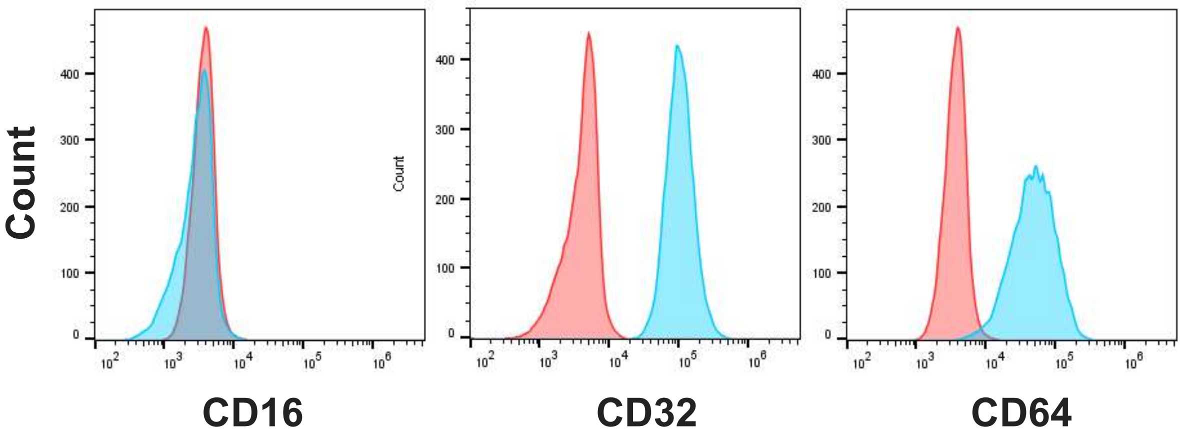

Supplemental Figure 1

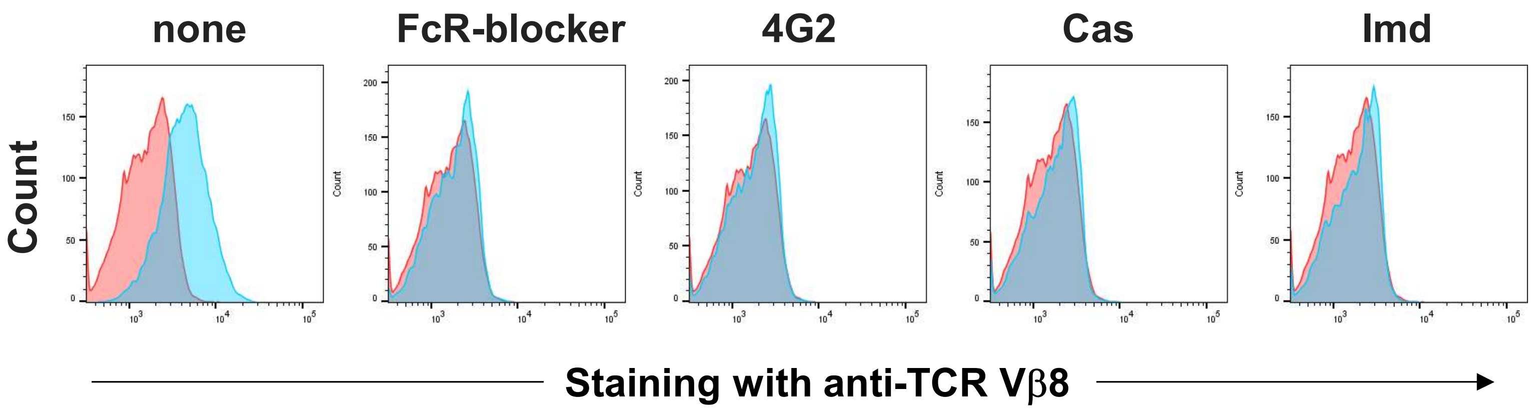

Supplemental Figure 2

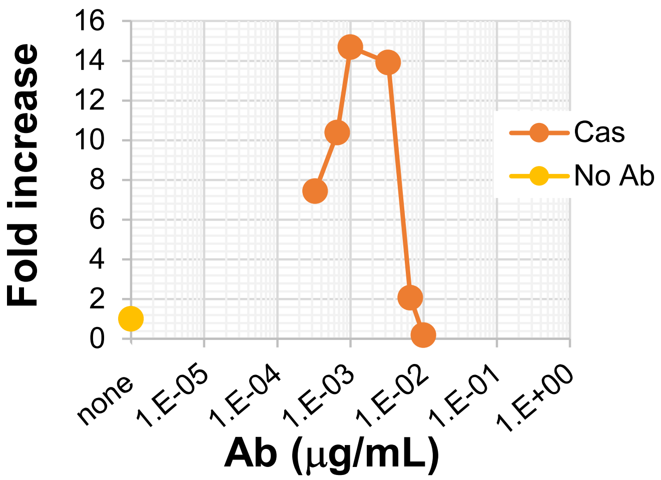

Supplemental Figure 3

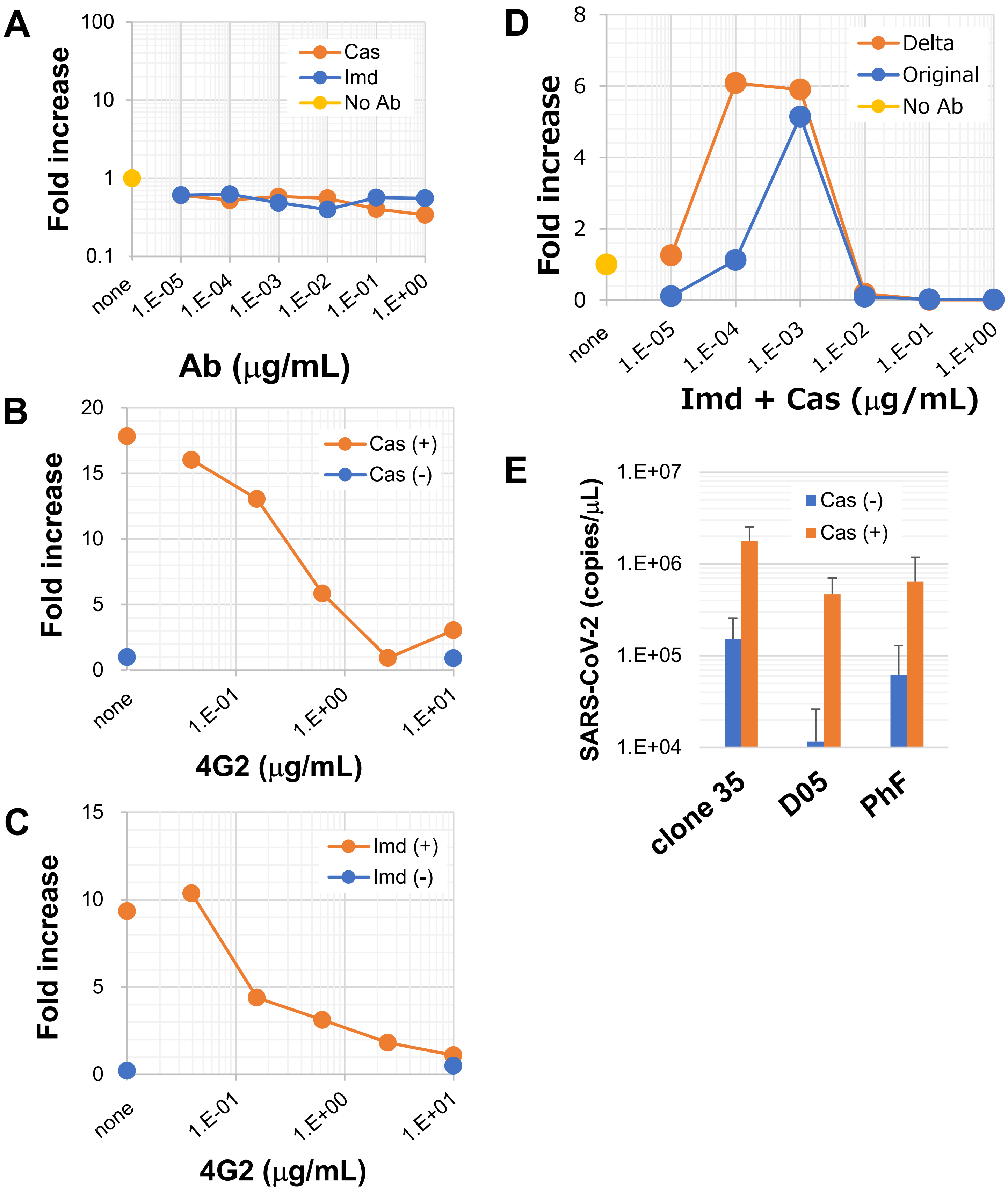

Supplemental Figure 4

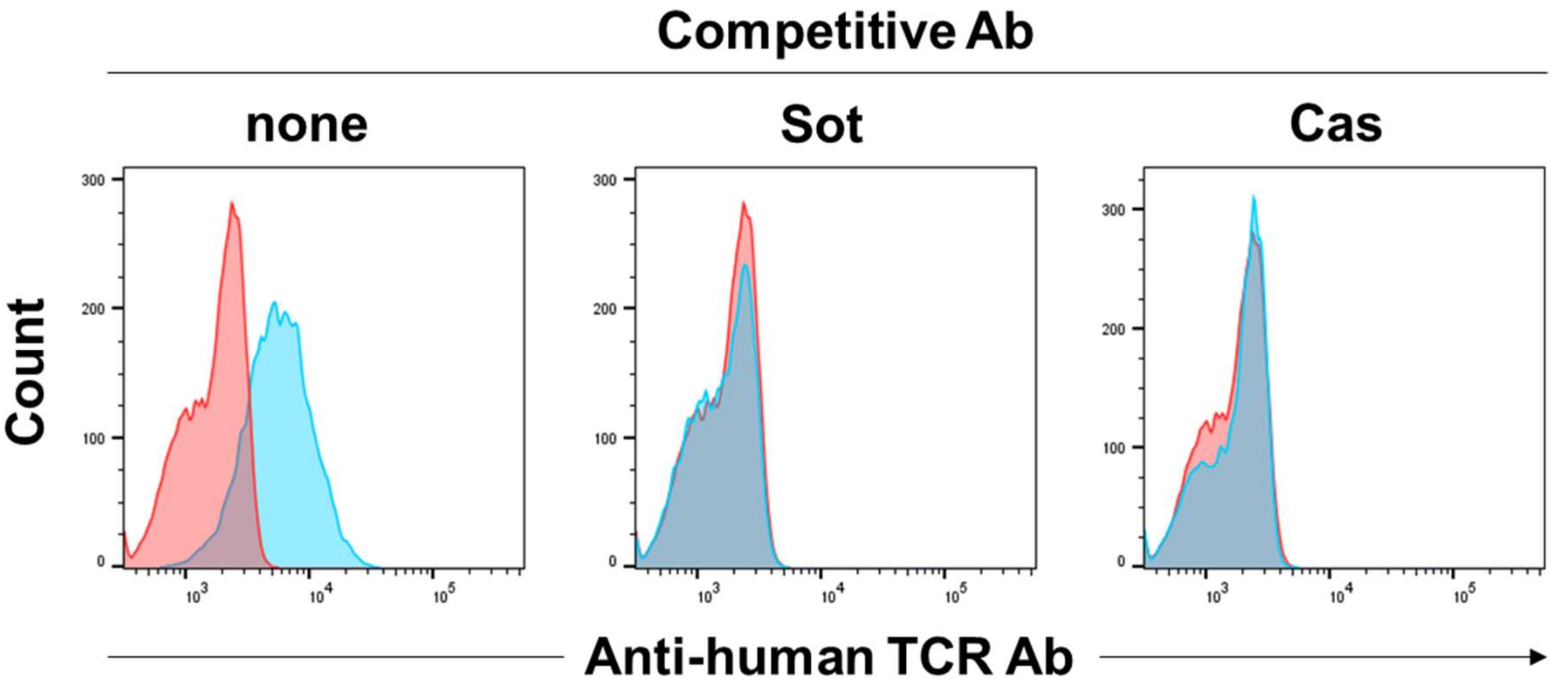

Supplemental Figure 5

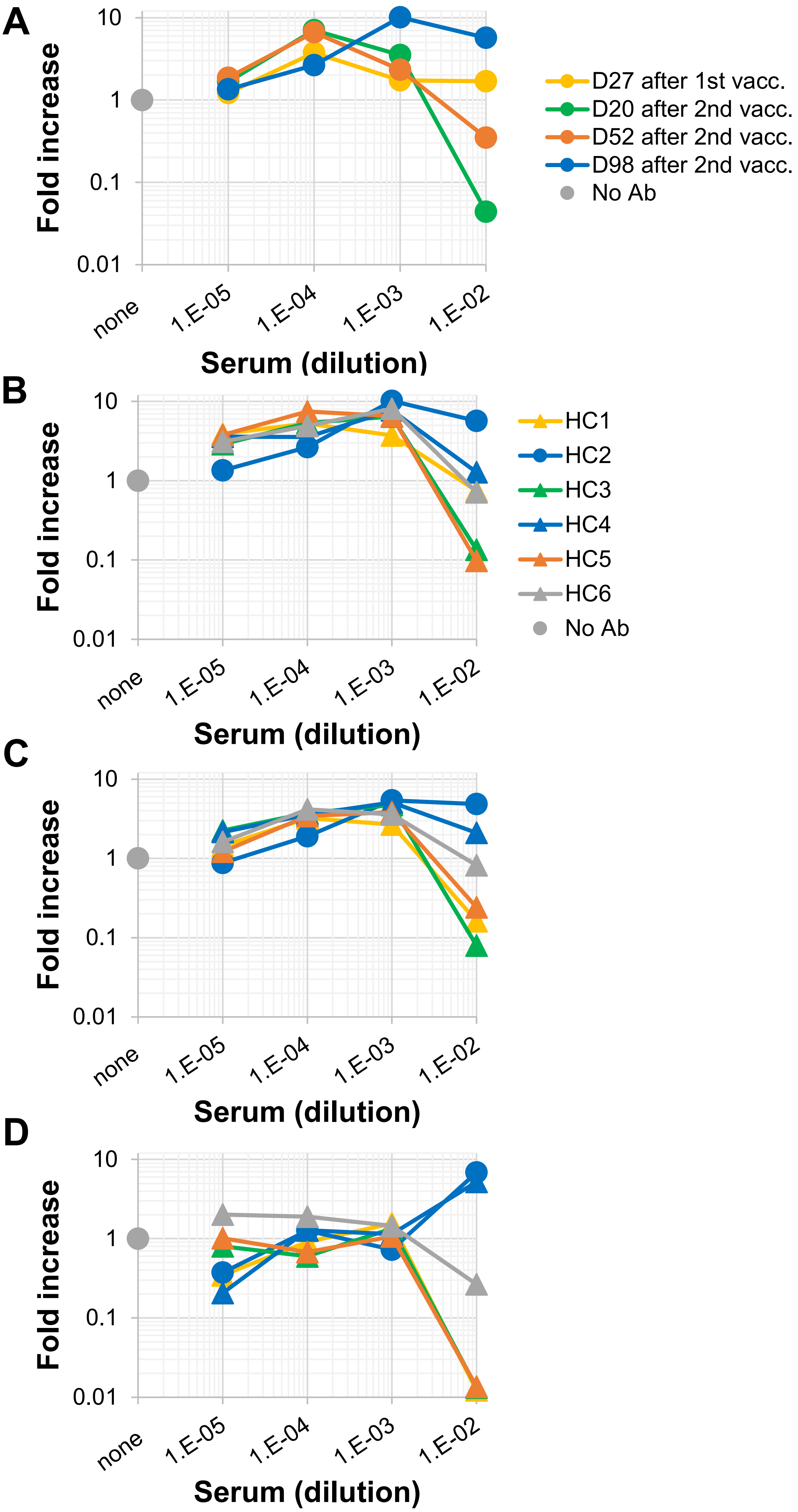

Supplemental Figure 6

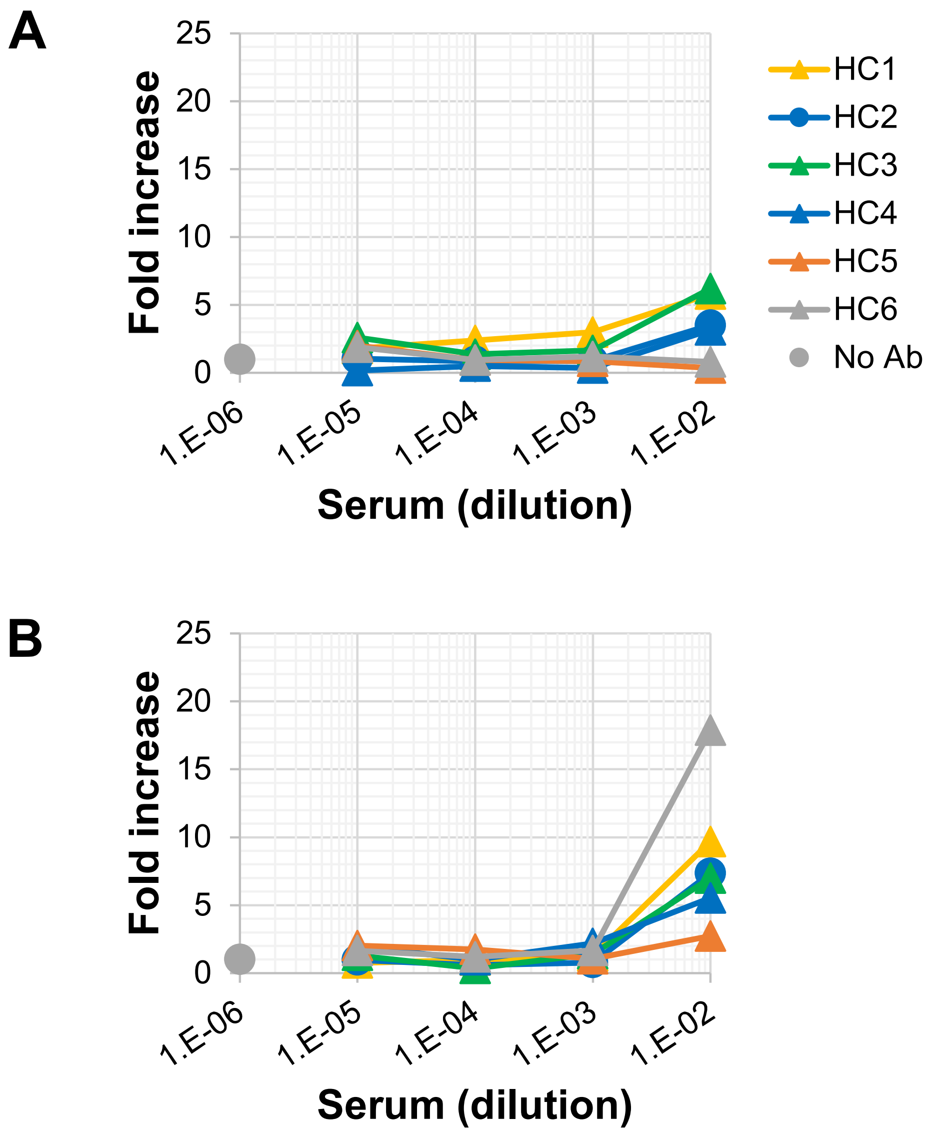

Supplemental Figure 7

A

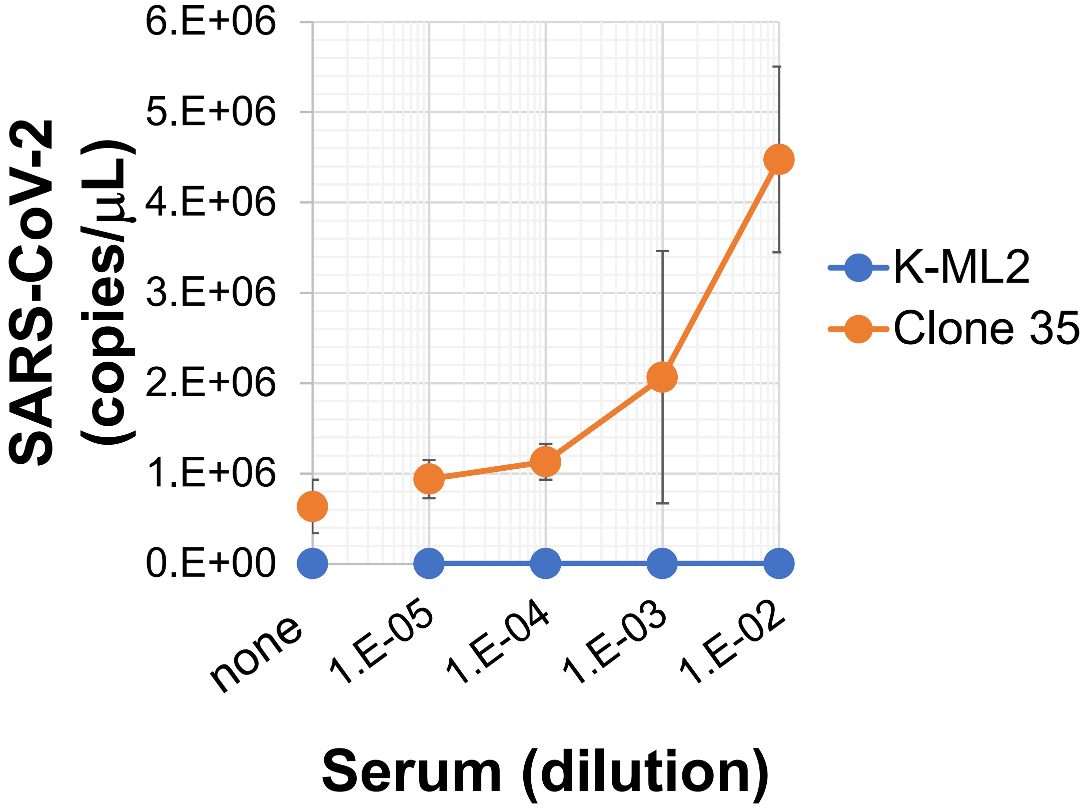

B

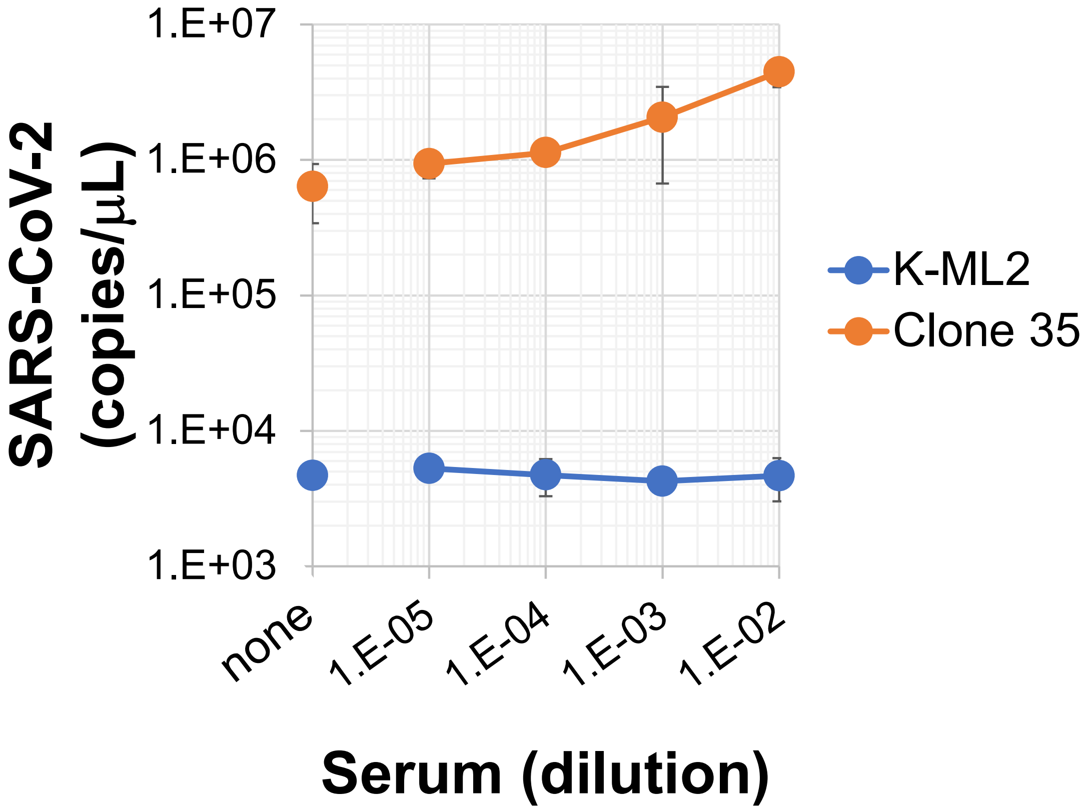

Supplemental Figure 8
